# Diversity and conservation of plant small secreted proteins associated with arbuscular mycorrhizal symbiosis

**DOI:** 10.1093/hr/uhac043

**Published:** 2022-02-19

**Authors:** Xiao-Li Hu, Jin Zhang, Rakesh Kaundal, Raghav Kataria, Jesse L Labbé, Julie C Mitchell, Timothy J Tschaplinski, Gerald A Tuskan, Zong-Ming (Max) Cheng, Xiaohan Yang

**Affiliations:** 1 Department of Plant Sciences, University of Tennessee, Knoxville, TN 37996, USA; 2 Biosciences Division, Oak Ridge National Laboratory, Oak Ridge, TN 37831, USA; 3 State Key Laboratory of Subtropical Silviculture, Zhejiang A&F University, Hangzhou, Zhejiang 311300, China; 4 Department of Plants, Soils and Climate, Utah State University, Logan, UT 84322, USA; 5 The Center for Bioenergy Innovation, Oak Ridge National Laboratory, Oak Ridge, TN 37831, USA; 6 College of Horticulture, Nanjing Agricultural University, Nanjing, Jiangsu 210095 China

## Abstract

Arbuscular mycorrhizal symbiosis (AMS) is widespread mutualistic association between plants and fungi, which plays an essential role in nutrient exchange, enhancement in plant stress resistance, development of host, and ecosystem sustainability. Previous studies have shown that plant small secreted proteins (SSPs) are involved in beneficial symbiotic interactions. However, the role of SSPs in the evolution of AMS has not been well studied yet. In this study, we performed computational analysis of SSPs in 60 plant species and identified three AMS-specific ortholog groups containing SSPs only from at least 30% of the AMS species in this study and three AMS-preferential ortholog groups containing SSPs from both AMS and non-AMS species, with AMS species containing significantly more SSPs than non-AMS species. We found that independent lineages of monocot and eudicot plants contained genes in the AMS-specific ortholog groups and had significant expansion in the AMS-preferential ortholog groups. Also, two AMS-preferential ortholog groups showed convergent changes, between monocot and eudicot species, in gene expression in response to arbuscular mycorrhizal fungus *Rhizophagus irregularis*. Furthermore, conserved *cis*-elements were identified in the promoter regions of the genes showing convergent gene expression. We found that the SSPs, and their closely related homologs, in each of three AMS-preferential ortholog groups, had some local variations in the protein structural alignment. We also identified genes co-expressed with the *Populus trichocarpa* SSP genes in the AMS-preferential ortholog groups. This first plant kingdom-wide analysis on SSP provides insights on plant-AMS convergent evolution with specific SSP gene expression and local diversification of protein structures.

## Introduction

Plant small secreted proteins (SSPs), defined as less than 250 amino acids (aa) in length, are derived from large precursors or encoded by small open reading frames (sORFs) [1–3]. SSPs play roles in many biological processes, including plant growth and development, response to various stresses, and mediation of intercellular communications [4, 5]. For example, in *Arabidopsis thaliana*, CLAVATA3/ESR-related protein 3 (CLE3) is involved in the regulation of lateral root formation [6]. Root-derived CLAVATA3/ESR-related protein 25 (CLE25) transmits water deficiency signals to leaves through vascular tissues in *A. thaliana* and ultimately increases dehydration tolerance [7]. Overexpression of C-terminally encoded peptide 1 (CEP1), another SSP, leads to inhibition of lateral root development and enhancement of nodulation in *Medicago truncatula* [8]. Over 90% of all plant species are associated with mycorrhizal fungi [9]. Legumes are particularly well studied species, with growing evidence suggesting that SSPs regulate legume growth, nodulation, and nutrient acquisition [10]. A recent study showed that a total of 417 putative plant SSPs were significantly regulated during the process of forming the mutualistic symbiosis between *Populus trichocarpa* roots and the ectomycorrhizal fungus *Laccaria bicolor*, indicating that plant-derived SSPs play potential roles in cross-kingdom interactions [11]. However, the functions of plant SSPs during mutualistic symbiosis remain largely unknown.

SSP discovery in plants typically starts by digitally mining sORFs in the sequenced plant genomes. With the affordability of genome sequencing and recent advances in transcriptomics, high-throughput identification of sORFs is becoming routine. Based on two commonly used metrics, sequence conservation and sequence similarity [12], multiple bioinformatics methods have been developed to aid the prediction of sORFs, such as sORF finder, which is an evolutionary selective constraints-based tool [13], and SPADA, which is a sequence homology-based software [14]. Furthermore, various tools have emerged for assessing the coding potential of putative sORFs, such as Coding-Non-Coding Identifying Tool (CNIT) based on support vector machine (SVM) [15], MiPepid based on a logistic regression model [16], and DeepCPP based on a deep neural network [17]. After generating sORF candidates, machine learning-based methods can be used for secretion prediction. Prediction of conventional secretion is primarily achieved by predicting N-terminal signal peptides through SignalP [18], and excluding proteins containing transmembrane regions, which can be predicted by TMHMM [19]. Then unconventional secretion of proteins that do not have N-terminal signal peptides can be predicted by SecretomeP [20], ApoplastP [21], BUSCA [22], and Plant-mSubP [23]. Finally, SSP candidates, which have complete sORFs, extracellular localization, and N-terminal signal peptides but without transmembrane regions, can be selected for functional characterization.

In the past five years, several pipelines, which combine several methods, have been used for SSP prediction. For example, a list of predicted novel SSPs in *M. truncatula* was created by using multiple sequential filtering steps, including protein length selection (<230 aa), signal peptide identification, and removal of proteins containing transmembrane helices and endoplasmic reticulum-retention signals [10]. In another study, discovery of SSPs in *P. trichocarpa* based on RNA-Seq datasets was achieved by selecting complete ORFs that encode proteins of less than 250 aa in length, followed by prediction of protein secretion using three different tools [11].

Arbuscular mycorrhizal symbiosis (AMS) is one of the most ancient and broadly occurring mutualistic associations between plants and arbuscular mycorrhizal fungi (AMF) [24]. This intimate relationship improves plant mineral nutrition acquisition, which potentially enhances crop yield [25]. In addition, AMS can increase plant tolerance to biotic and abiotic stresses [25–28]. AMS also contributes to many ecosystem functions, including soil aggregation, less fertilizer utilization, and reduction of nutrient losses [29]. Over the past two decades, based on the alteration of symbiosis phenotypes in gene knockout or knockdown mutants, a number of genes have been identified to be involved in AMS [24]. Recently, with the availability of rich plant genomic resources, phylogenomic analyses have provided a great opportunity for studying the evolutionary pattern of conserved genes in plants in relation to AMS [30]. Recently, the expression of two SSP genes, *LjCLE19* and *LjCLE20* in *Lotus japonicus*, was regulated by AMF *Rhizophagus irregularis* [31]. More recently, some putative sORF-encoding genes in *Populus* were reported to be responsive to *R. irregularis* [32].

The goal of this study was to gain a better understanding of the relationship between plant SSPs and AMS. To achieve this goal, we predicted SSPs with 50–250 amino acids in length from 60 sequenced plant genomes using a customized computational pipeline and identified candidate plant SSP genes that are potentially involved in AMS through phylogenetic analysis of ortholog groups containing SSP genes and identification of gene expression responsive to AMF. Furthermore, we performed comparative analysis of 3D-protein structure and the promoter regions between genes in selected ortholog groups, which were either specific to or predominately represented by AMS plant species. Finally, we built co-expression networks using *P. trichocarpa* genes to identify other genes associated with the *P. trichocarpa* SSP genes in the ortholog groups predominately represented by AMS plant species. Our results indicate that convergency in SSP sequences and gene expression induced by fungi is related to convergent emergence of AMS in diverse plant species. The SSP candidates identified in this study lay the foundation for experimental characterization of AMS-related genes to gain a deeper understanding of the molecular mechanisms underlying the interactions between plants and AMF.

## Results

### Identification of small secreted proteins (SSPs) in 60 plant species

**Figure 1:**
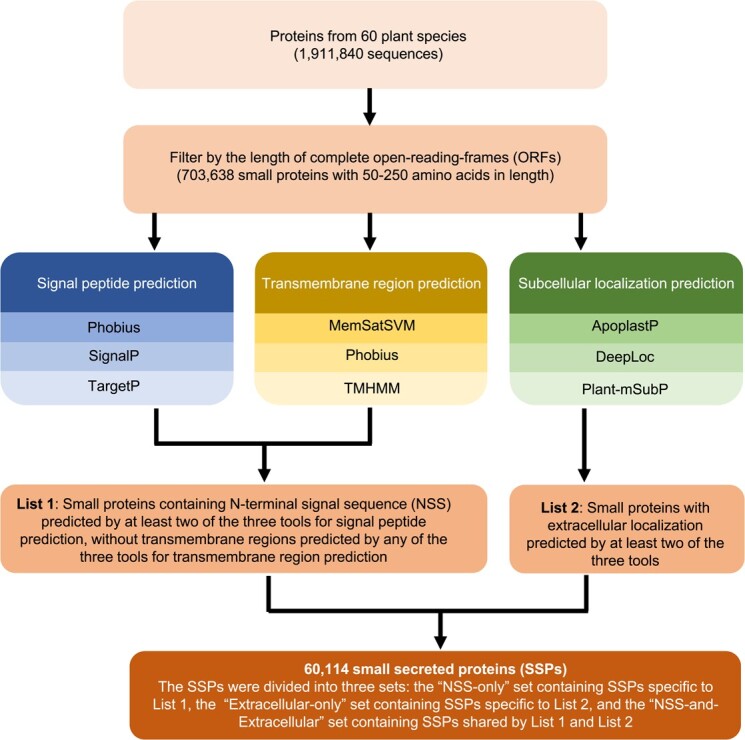
A computational pipeline used for predicting small secreted proteins (SSPs) in plant genomes. The input was the primary protein sequences of 60 plant species listed in Table S1. Small proteins with a full-length of 50–250 aa were identified for secretion prediction using different methods. Conventional protein secretion featured by N-terminal signal sequence (NSS) were predicted by using SignalP 5.0 [16], Phobius [57], and TargetP [58]. Transmembrane domains were identified by using TMHMM 2.0 [17], MEMSAT-SVM [59], and Phobius [57]. Extracellular protein localization was predicted by using ApoplastP [19], DeepLoc [60], and Plant-mSubP [21]

**Figure 2:**
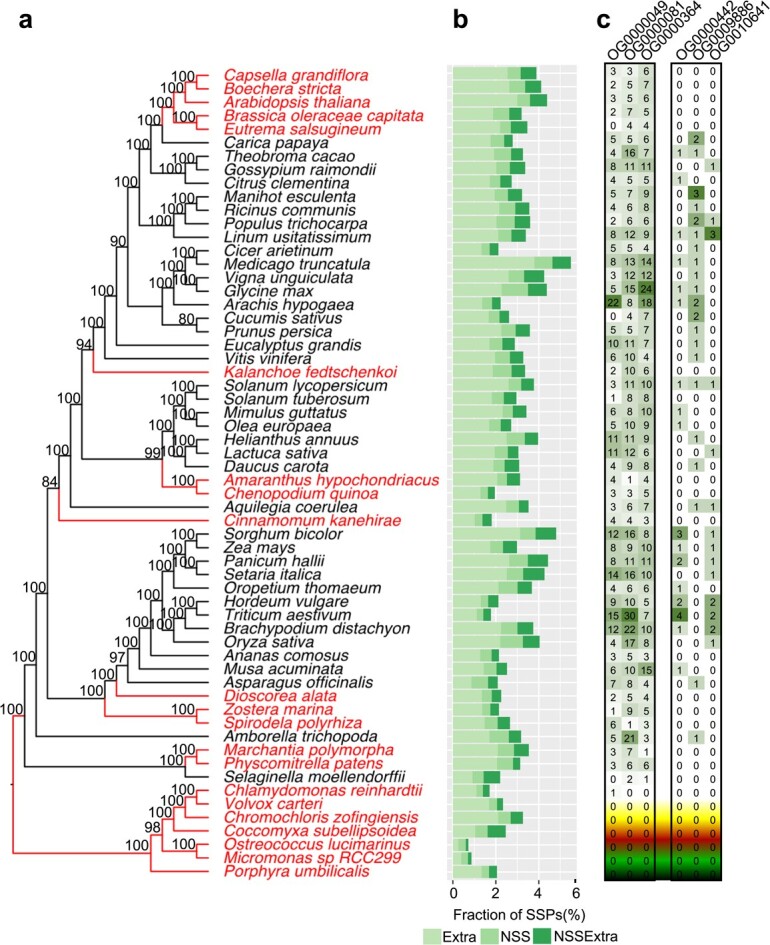
A coalescent-based maximum likelihood phylogenetic tree of 60 plant species inferred from single copy gene trees. (**a**) Bootstrap support values of 50% or higher were shown on the phylogenetic tree. Plant species with and without the ability to form AMS are indicated in black and red, respectively. (**b**) The bar plot on the right side of the phylogenetic tree indicates the fraction of predicted SSPs in each plant species. Extra represents SSPs in the “Extracellular-only set”; NSS represents SSPs in the “NSS-only set”; and NSSExtra represents SSPs in the “NSS-and-extracellular set”, as defined in Fig. 1. (**c**) Number of small secreted proteins (SSPs) in representative ortholog groups. OG0000049, OG0000081, and OG0000364 are AMS-preferential ortholog groups containing significantly (*P* < 0.05) more SSPs from the AMS species than from the non-AMS species. OG0000442, OG0009886, and OG0010641 are AMS-specific ortholog groups containing SSPs from at least 30% of the 39 AMS species but not from any non-AMS species. Relative abundances of SSPs within each ortholog group are represented by a color scale.

To investigate the distribution of SSPs in plant genomes, a total of 60 plant species, including 33 dicots, 15 monocots and 12 more ancient plant species listed in Table S1, were used to predict two lists of SSPs using a computational pipeline illustrated in Fig. 1. The first SSP list included 23 360 SSPs containing N-terminal signal sequence (NSS), without transmembrane regions (Figs. S1a and S1b). The second SSP list contained 48 081 SSPs with extracellular localization predicted by at least two methods (Fig. S1c). By combining these two SSP lists, we generated a non-redundant list of 60 114 SSPs (Table S3), which were divided into three sets: (i) the NSS-only set containing 12 033 SSPs from the first SSP list only, (ii) the Extracellular-only set containing 36 754 SSPs from the second SSP list only, and (iii) the NSS-and-extracellular set containing 11,327 SSPs shared by the two SSP lists (Fig. S1d). The distribution of SSP numbers in each plant species was illustrated in Fig. 2b. Also, we found that there were significant (*P* ≤ 0.05, Wilcoxon-rank sum test) differences in both the number and the ratio (i.e. the number of SSPs divided by the total number of proteins annotated in a genome) of SSPs between AMS and non-AMS species (Figs. S2a and S2b). Similar significant (*P* ≤ 0.05, Wilcoxon-rank sum test) difference between AMS and non-AMS species was found only in the number of SSPs in monocot lineage but not in dicots and other species (i.e. more ancient plant species) (Figs. S2c and S2d).

### AMS-related ortholog groups

We identified 60 981 ortholog groups accounting for 91.6% of total number of protein sequences from 60 plant species listed in Table S1, including 39 AMS species and 21 non-AMS species. Among these, 9390 ortholog groups contain 49 472 predicted SSPs, which account for 82.3% of total number of SSPs predicted from the 60 plant species. Among the SSP-containing ortholog groups, 6629 ortholog groups contained SSPs from AMS plant species only, 1817 ortholog groups contained SSPs from non-AMS plant species only, and 944 ortholog groups contained SSPs from both AMS and non-AMS species. Aiming to identify ortholog groups that are highly associated with AMS status, we first selected three AMS-specific ortholog groups containing proteins from at least 30% of the 39 AMS-associated species but not from any non-AMS species, including OG0000442 (containing genes encoding heavy-metal associated domain proteins), OG0009886 (containing genes encoding wall-associated receptor kinase), and OG0010641 (containing genes encoding proteins with unknown function). Then, from the ortholog groups containing SSPs from both AMS and non-AMS species, we identified three AMS-preferential ortholog groups (APOGs), in which the number of SSPs from the AMS species was significantly (*P* ≤ 0.05) higher than that from the non-AMS species, including OG0000049 (containing genes encoding plastocyanin-like proteins and glycosylphosphatidylinositol-anchored proteins (GPI-APs)), OG0000081 (containing genes encoding Dirigent proteins), and OG0000364 (containing genes encoding EPFL proteins). These AMS-specific and AMS-preferential genes were not found in the ancient plant lineages, such as *Chromochloris zofingiensis*, *Chlamydomonas reinhardtii* and *Porphyra umbilicalis*. The observed repeated emergences or expansion in such multiple, non-neighboring plant lineages (Figs. 2a and c), suggests that these AMS-associated genes resulted from convergent evolution.

### AMF-regulated gene expression

**Figure 3:**
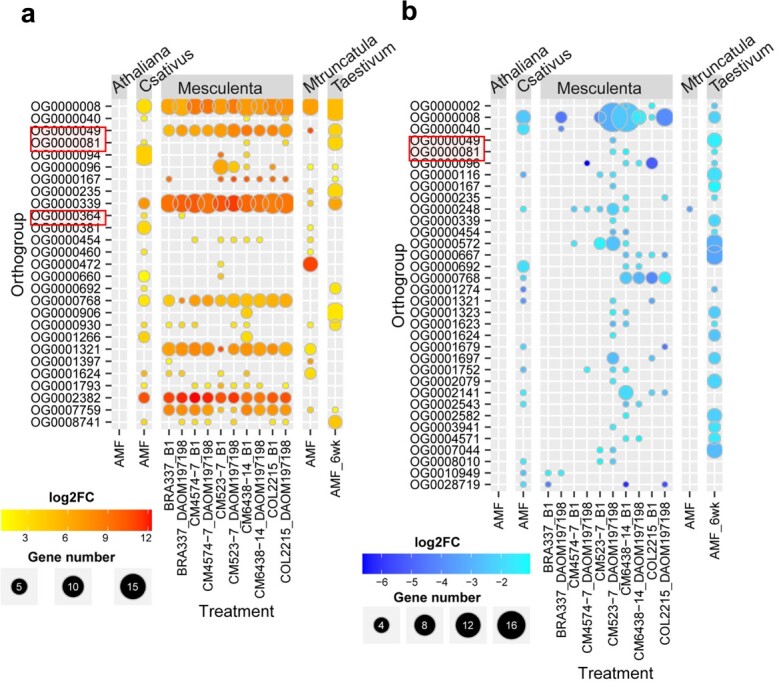
Ortholog groups containing small secreted proteins (SSPs) showing differential gene expression in response to AMF *Rhizophagus irregularis* in at least two plant species. (**a**) Upregulation of plant SSP gene expression by the AMF treatment. (**b**) Downregulation of plant SSP gene expression by the AMF treatment. The heatmap represents log2(fold change) of transcript abundance between AMF treatment versus control and the circle size indicates the number of SSPs in each ortholog group. The differential gene expression between AMF treatment and control was defined as at least two-fold change in transcript abundance, along with adjusted *P* < 0.05.

To identify AMF-inducible SSPs, we performed a cross-species comparative analysis of gene expression in four AMS plant species (*Cucumis sativus*, *Manihot esculenta*, *M. truncatula*, and *Triticum aestivum*) and one non-AMS plant species (*A. thaliana*), which were inoculated with AMF *R. irregularis* (Table S2). Through analysis of differential gene expression between AMF treatments and corresponding controls at different time points after fungal inoculation, we identified a total of 45, 3255, 8582, 1263, and 8205 differentially expressed genes (DEGs) in *A. thaliana*, *C. sativus*, *M. esculenta*, *M. truncatula*, and *T. aestivum*, respectively (Table S4). To further explore if the expression of SSPs were affected by AMF, we checked the DEGs encoding SSPs in these species. We identified 91, 330, 47, and 193 differentially expressed SSPs in *C. sativus*, *M. esculenta*, *M. truncatula*, and *T. aestivum*, respectively. No differentially expressed SSPs were found in non-AMS *A. thaliana* (Table S5). Furthermore, we identified 27 and 34 ortholog groups containing SSPs that were up- and down-regulated, respectively, by AMF treatment in at least two out of the four AMS species (Fig. 3), suggesting convergency in AMF-responsive gene expression among different plant species.

### Diversification and conservation among genes in the AMS-preferential ortholog groups containing AMF-inducible SSPs

**Figure 4:**
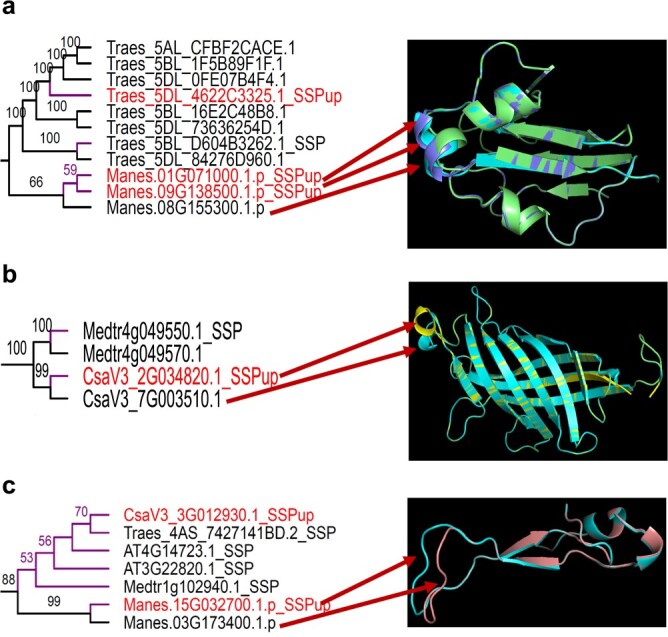
Structure modelling of AMS-related small secreted proteins (SSPs) and their closely related non-SSP sequences in the AMS-preferential ortholog groups. Different colors indicate different proteins. Red arrows point out local variations found in protein structures in the AMS-preferential ortholog groups OG0000049 (**a**), OG0000081 (**b**), and OG0000364 (**c**).

We applied 3D protein structural prediction for AMF-inducible SSPs, and their closely related non-SSPs in the phylogenetic trees (Figs. S3, S4 and S5) of AMS-preferential ortholog groups, to determine divergence of protein functions [33]. We selected gene clusters containing genes that encode AMF-inducible SSPs, and predicted 3D structures of 11, 4, and 7 proteins in ortholog groups OG0000049, OG0000081, and OG0000364, respectively. We found that the AMF-inducible SSPs, and their closely related homologs, in each of three ortholog groups, had the same general fold according to homology-based structural modeling, with some local variations in the protein structural alignment, as exemplified by the regions marked by the red arrows (Fig. 4). We acknowledge that 3D protein structural predictions will not show the maximum difference among protein pairs, and the exact structures and alignments however may vary more than what the homology-based modeling data might suggest. Still, the 3D protein structural predictions facilitate comparative examinations.

**Figure 5:**
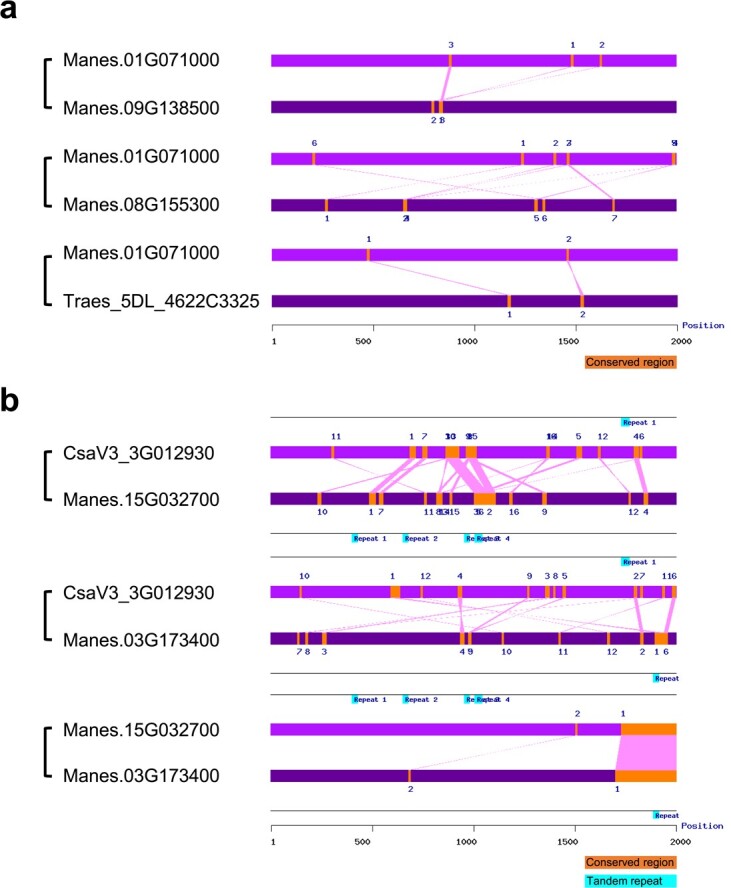
Promoter alignment between different gene pairs selected from AMS-preferential ortholog groups. Conserved blocks were located in the promoter regions (i.e. 2000 bp upstream of the translation start codon) of AMF-inducible small secreted protein (SSP) genes, in comparison with closely related non-SSP genes, which are selected from AMS-preferential ortholog groups OG0000049 (**a**) and OG0000364 (**b**).

It is generally known that conserved *cis*-acting elements located in the gene promoter region regulate gene expression patterns [33]. We conducted a comparative analysis of promoter sequences (i.e. 2000 bp upstream of the translation start codon) between various gene pairs selected from two AMS-preferential ortholog groups. Four genes, including three AMF-inducible SSPs (Manes.01G071000, Manes.09G138500, and Traes_5DL_4622C3325) and one closely related homolog (Manes.08G155300), were selected from OG0000049 as representatives. In addition, three genes, including two AMF-inducible SSPs (CsaV3_3G012930, and Manes.15G032700) and one closely related homolog (Manes.03G173400), were selected from OG0000364 (Fig. S6). Consequently, three *cis*-acting elements were found to be conserved in the promoter regions of SSP genes upregulated by AMF, including the binding sites of transcription factors bHLH, GATA, and MYB, (Fig. 5), which have been reported to be involved in response to abiotic stresses, cell wall modification, and pathogens, respectively [34–36].

### Co-expression analysis

**Figure 6:**
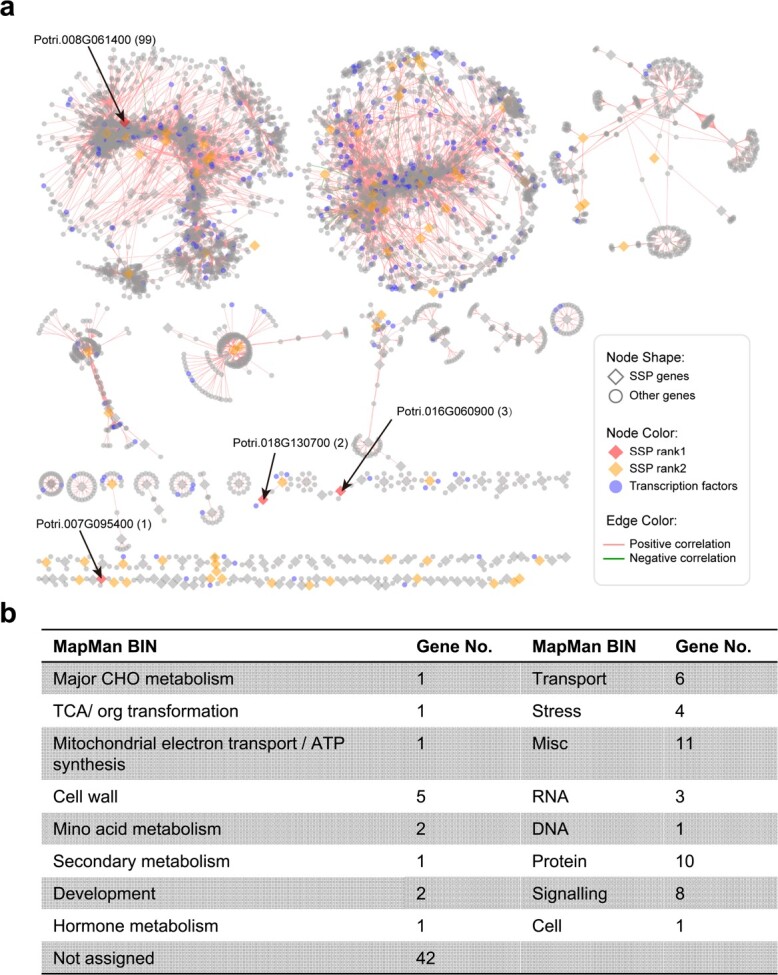
Co-expression network of *Populus trichocarpa* small secreted proteins (SSPs) in AMS-specific ortholog groups, AMS-preferential ortholog groups, and ortholog groups containing differential expressed SSPs from at least two species. **(a)** “SSP rank1” represents SSPs shared by the AMS-preferential ortholog groups and the ortholog groups containing differential expressed SSPs from at least two species in response to AMF *Rhizophagus irregularis*. “SSP rank2” represents SSPs from the AMS-specific ortholog groups or the AMS-preferential ortholog groups or the ortholog groups containing differential expressed SSPs from at least two species in response to AMF. (**b**) Functional classification of 99 co-expressed genes of Potri.008G061400 is presented.

To uncover additional context for the potential function and evolutionary divergence of SSPs, the SSP co-expression networks were constructed using the woody model plant *P. trichocarpa* as an example, which is an AMS plant species with a large amount of public gene expression datasets available, covering various plant tissues, different plant developmental stages and response to several stresses. To uncover the high confidential co-expression relationships, we extracted the highly co-expressed genes (|PCC| ≥ 0.95) based on the *P. trichocarpa* gene atlas. From 1248 SSPs in *P. trichocarpa*, 353 SSPs were highly co-expressed with 34 980 genes. We then focused on the subnetworks of SSPs in the AMS-preferential ortholog groups (i.e. OG0000049, OG0000081, OG0000364). Four genes (Potri.008G061400, Potri.016G060900, Potri.018G130700, and Potri.007G095400) in OG0000081 and OG0000364 were co-expressed with 99, 3, 2 and 1 genes, respectively (Fig. 6). The gene set co-expressed with Potri.008G061400, which encodes a disease resistance-responsive/Dirigent-like protein, was overrepresented by genes involved in signaling, cell wall and stress (8, 5 and 4 genes, respectively), suggesting that Potri.008G061400 plays a role in diverse biological processes.

## Discussion

With the increasing number of sequenced plant genomes and advancement in bioinformatics, an increasing number of SSPs have been identified in numerous plant species. However, there are several limitations in previous studies on SSP prediction. First, much attention has been paid on predicting NSS-containing SSPs, overlooking SSPs associated with unconventional secretion pathways. Second, most of previous efforts have relied upon single computational methods for predicting protein secretion, resulting in biased results, because there is a big difference in the prediction result among different computational tools for protein secretion prediction (Figs. S1a, S1b and S1c). To reduce the false positive prediction of SSPs, we created a stringent workflow (Fig. 1) to predict SSPs, based on the consensus prediction of at least two of the three popular methods for predicting protein signal peptides or extracellular localization. This computational pipeline would be useful for predicting new SSPs in more plant species when new plant genome sequence data become available in the future. Also, proteomics-based secretome data are very useful for consolidating the computational prediction of SSPs based on the analysis of protein sequences [25]. However, to our knowledge, currently there are no proteomics-based secretome data available for plant species under AMF treatments. Therefore, it is important to perform proteomic analysis of samples enriched with secreted proteins in plants treated by AMF strains to establish a foundation for future study on plant-AMF interaction at the secretome level.

Convergent evolution plays an important role in plant-microbe interactions [37–39]. Our phylogenomic analysis revealed that AMS emerged in multiple plant lineages through convergent evolution (Fig. 2a). We found that some of the SSP genes, in the AMS-preferential ortholog groups OG0000049 and OG0000364, showed convergent change (i.e. up-regulation) in transcript abundance, between eudicot and monocot plant species, in response to AMF *R. irregularis* (Fig. 4a and 4c). It can be hypothesized that the convergent change in SSP gene expression has contributed to the independent emergence of AMS in eudicot and monocot plants lineages. This hypothesis could be tested by examining whether over-expression of the AMF-inducible SSP genes can enhance the beneficial interactions between the AMS plants and their symbiotic partner *R. irregularis* and promote the initiation of symbiosis between *R. irregularis* and the non-host plant species. Similar approach has been successfully used to investigate the role of a *P. trichocarpa* G-type lectin receptor-like kinase PtLecRLK1 in the initiation of symbiosis between ectomycorrhizal fungus *L. bicolor* and two non-host plant species (*A. thaliana* and *Panicum virgatum*) [40, 41]. Also, the powerful clustered regularly interspaced short palindromic repeats (CRISPR)/CRISPR-associated system (Cas) [42, 43] could be used for generating loss-of-function mutants to validate the function of the plant SSP genes predicted in this study. A plant biological process may involve a gene module that contains multiples genes [44]. To study the potential collective effect of multiple SSPs on AMS, multiplex CRISPR constructs [45] can be designed to target multiple candidate SSP genes identified in this study. Due to the limited availability of public RNA-Seq data, we have analyzed the expression of SSP genes in four AMS plant species and one non-AMS plant species in response to one AMF species (i.e. *R. irregularis*). In the future, it is necessary to generate RNA-Seq data for more AMS and non-AMS plant species under the treatment by additional AMF species besides *R. irregularis* so that the following two important questions can be addressed. First, can the plant SSPs responsive to *R. irregularis* play a role in the symbiosis between plants and other AMF species? Second, are there any AMF-inducible SSP genes in the three AMS-specific ortholog groups (i.e. OG0000442, OG0009886, OG0010641)? We can assume that answering these questions based on the analysis of additional RNA-Seq data would provide a deeper understanding of the role of SSPs in the evolution of AMS.

Our comparative analysis uncovered some *cis*-elements (e.g. transcription factor binding sites) conserved in the promoter regions of the AMF-inducible SSP genes between eudicot and monocot species (Fig. 5). To gain a deep understanding of transcriptional regulation of the AMF-inducible SSP genes, future experimental work will be needed to identify the transcription factors that bind to the *cis*-elements. Also, our protein structural modeling revealed some local variations in the protein structural alignment between the SSPs, and their closely related homologs, in each of the three AMS-preferential ortholog groups (Fig. 5). Future research will be needed to examine whether these differences in protein structure contribute to the functional diversification between AMF-inducible SSPs and their closely related homologs. For example, one strategy could be to investigate the impact of swapping the polymorphic protein sequence regions between the AMF-inducible SSPs and their closely related homologs in transgenic plants on the interactions between plants and their symbiotic fungal partners.

The SSP genes in ortholog group OG0000049 encode GPI-APs, which are ubiquitous and abundant among eukaryotes [46]. To date, more than 300 GPI-APs have been identified in *A. thaliana*. These proteins are involved in signaling transduction during multiple biological processes, such as cell wall composition, hormone signaling responses, and pathogen responses [47]. In addition, we found that several SSP genes encoding disease resistance-responsive proteins in ortholog group OG0000081 were upregulated by AMF. It has been reported that both symbiotic and pathogenic processes can share the same signaling pathways [48]. Therefore, we hypothesize that SSPs play an important role in plant response to both pathogenic and beneficial microbes. Future experimental research will be needed to investigate the potential impact of the SSP genes in ortholog groups OG0000049 and OG0000081 on AMS as well as other biological processes such as pathogen responses.

Poplar (*Populus* spp.) is an important woody crop for bioenergy, horticulture, and ecosystem services [49, 50]. *P. trichocarpa* can form mutualistic symbiosis with not only AMF but also ectomycorrhizal fungus. AMF facilitates plant uptake of nutrients such as phosphate, helping plants to adapt to phosphate-limiting environments [51]. Several studies have shown that SSPs either produced from fungi or poplar affected the growth of the fungi, consequently contributing to host colonization and nutrient transfer [11, 52]. There are a large number of gene expression datasets available for this model tree species in public databases. Based on the co-expression networks, we identified four *P. trichocarpa* genes (i.e. Potri.008G061400, Potri.016G060900, Potri.018G130700, and Potri.007G095400) in two AMS-preferential ortholog groups, which were co-expressed with other genes. For example, Potri.008G061400 encoding a disease resistance-responsive/Dirigent-like protein is co-expressed with 99 genes with diverse functions (Fig. 6b). Dirigent proteins have been found to modulate cell wall metabolism during adaptive responses, along with their involvement in lignan and lignin biosynthesis, which are associated with plant development [53]. This result suggests that SSPs could function in a complex network regulating multiple biological processes. Currently our understanding of how plant SSPs regulate symbiosis is limited. More in-depth research and experimental validation on how poplar SSPs affect plant-fungal symbiosis should be conducted in the future.

In summary, this study established a computational pipeline for genome-wide prediction of SSPs in plants and identified some plant SSP candidates that are potentially involved in AMS. Furthermore, our comparative analysis revealed convergent changes in SSP gene expression and gene regulatory elements between monocot and eudicot species, as well as diversification of protein structure between AMF-inducible SSPs and their closely related homologs, suggesting that SSPs might have played an important role in the AMS evolution in plants.

## Materials and methods

### Plant species and protein sequences

Primary protein sequences (i.e. the longest protein sequence for each gene) were downloaded from Phytozome13 (https://phytozome-next.jgi.doe.gov) for a total of 60 plant species representing diverse plant lineages (Table S1), including dicot, monocot, basal angiosperm, bryophyte, lycophyte, chlorophyta, and rhodophyta. Symbiosis status of plant species was determined based on the published literature [54, 55].

### Construction of ortholog groups and phylogenetic trees

The primary protein sequences of the 60 plant species were used as input to construct ortholog groups using Orthofinder [56]. For constructing gene trees, the protein sequences of each mostly single-copy orthologue group, which contains no more than 3 genes in each plant species, aligned using MAFFT version7 [57]. The protein sequence alignments were further trimmed by removing sites with more than 50% gaps or Ns and removing sequences less than 50% of the alignment in length. The trimmed protein sequence alignments were used to create gene trees using the maximum likelihood approach implemented in IQ-Tree 2 [58] (default parameters; 1000 bootstrap replications), with the best-fitting substitution models determined by ModelFinder [59]. Then, the species tree was generated from the gene trees by performing coalescent-based analysis using ASTRAL [60].

### Prediction of small secreted proteins (SSPs)

We created a computational pipeline to predict SSPs from a total of 1 911 840 protein sequences in 60 plant genomes (Fig. 1). Briefly, small proteins (encoded by complete ORFs with both start and stop codons) of 50–250 amino acids in length were selected as an initial small protein subset. The secretion prediction for proteins in the initial small protein subset was performed using eight either widely used or recently released methods based on different algorithms. Specifically, SignalP 5.0 [18], Phobius [61], and TargetP [62] were used for the prediction of N-terminal signal sequence (NSS). TMHMM 2.0 [19], MEMSAT-SVM [63], and Phobius [61] were used for the prediction of transmembrane domains. ApoplastP [21], DeepLoc [64], and Plant-mSubP [23] were used for the prediction of protein subcellular locations. Stand-alone applications of these selected methods were run on a computer cluster. The principle of majority-decision called MDSEC, as previously described [65], was used to predict SSPs (i.e. small proteins containing NSS predicted by at least two out of the three approaches, including SignalP 5.0 [18], Phobius [61], and TargetP [62], were considered to be secreted proteins). As NSS can also be found in membrane proteins, small proteins containing at least one transmembrane region predicted by each single tool were eliminated from the pool of predicted NSS-containing SSPs, resulting in the first list of predicted NSS-containing SSPs without transmembrane regions. In addition, a number of proteins without NSS can be secreted via unconventional secreted pathway [25]. Thus, we generated the second list of SSPs with extracellular location predicted by two out of the three approaches, including ApoplastP [21], DeepLoc [64], and Plant-mSubP [23]. Finally, a set of non-redundant predicted SSPs were generated by merging the first and the second lists of predicted SSPs mentioned above, which were further divided into three sub-categories: NSS-only (from the first list only), NSS-and-extracellular (shared by both the first and the second lists), and Extracellular-only (from the second list only).

### RNA-Seq data analysis

We performed a cross-species comparative transcriptome analysis using public RNA-Seq data of different plant roots inoculated with AMF, which include four AMS species, including *C. sativus, M. esculenta, M. truncatula*, and *T. aestivum*, as well as one non-AMS species *A. thaliana* as a control (Table S2). The raw reads retrieved from the National Center for Biotechnology Information Sequence Read Achieve (http://www.ncbi.nlm.nih.gov/sra/) were filtered with the BBDuk program from JGI’s BBTools (https://jgi.doe.gov/data-and-tools/bbtools) to trim adapters and extremities with a quality value per base lower than 20. After trimming adapter sequences and filtering out low-quality reads, the clean reads were mapped to the latest genome assembly for each species using STAR2.7.9a [66]. The mRNA abundance of each gene in each species was quantified as FPKM. Differentially expressed genes (DEGs) in each species were determined by applying EBSeq [67] in the R package. The cut-off for significant DEGs was an absolute log2(fold change) >1 and a false discovery rate (FDR) corrected *P*-value ≤0.05.

### Promoter analysis

The promoter sequences (i.e. 2000 bp upstream of the translation start codon) of the SSP genes that were upregulated by AMF, along with their closely related genes in the AMS-preferential ortholog groups were downloaded from Phytozome (https://phytozome-next.jgi.doe.gov). Conserved *cis*-elements in the promoter regions of AMF-inducible SSP genes were identified using the online server PlantPAN 3.0 [68] with default parameters.

### Protein structural modeling

The 3D structures of SSPs and their closely related proteins in the AMS-preferential ortholog groups were predicted using the Phyre2 web portal [69]. The protein structural alignments were constructed and visualized using PyMol (https://pymol.org/2/).

### Co-expression network analysis

For co-expression network construction, the expression data were obtained in the *Populus* Gene Atlas Study from Phytozome (https://phytozome-next.jgi.doe.gov). Pearson correlation coefficients (PCCs) were calculated between all pairs of genes. A threshold of *P*-value ≤0.05 and absolute PCC ≥ 0.95 were applied to identify the significant correlations, and their co-expression relationships were visualized by Cytoscape [70]. Functional classification of the co-expressed genes of candidate SSPs was carried out with MapMan [71].

## Supplementary Material

Web_Material_uhac043

## Data Availability

All data supporting this research result can be obtained in the paper and within Supplementary information published online.
