# Supplementary material for: Diversity and conservation of plant small secreted proteins associated with arbuscular mycorrhizal symbiosis
Source: Hortic Res. 2022 Feb 19;9:uhac043. doi: 10.1093/hr/uhac043 (PMC8985099; doi:10.1093/hr/uhac043)
Supplement: Web_Material_uhac043 [file web_material_uhac043.zip › Supplementary_figures/Supplementary_Figure1.pdf]

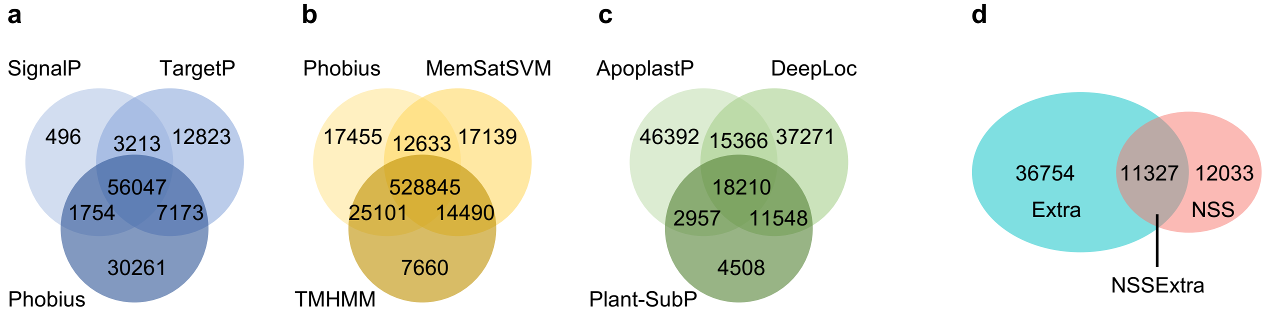

Supplementary Fig 1. Number of SSPs predicted by different tools. Venn diagrams show that number of proteins containing signal peptide predicted by using SignalP, TargetP and Phobius. The overlap gives the number of proteins with putative signal peptide predicted by two and three tools (**a**), and number of proteins without transmembrane regions predicted by using Phobius, MemSatSVM and TMHMM. The overlap gives the number of non-membrane spanning proteins predicted by two and three tools (**b**), and number of proteins with extracellular localization predicted by using ApoplastP, DeepLoc, Plant-SubP. The overlap gives the number of proteins with localization in extracellular space predicted by two or three tools (**c**), and the number of SSPs were characterized as Extracellular-only, NSS-only and NSS-plus-Extracellular **(d)**.
