# Supplementary material for: Diversity and conservation of plant small secreted proteins associated with arbuscular mycorrhizal symbiosis
Source: Hortic Res. 2022 Feb 19;9:uhac043. doi: 10.1093/hr/uhac043 (PMC8985099; doi:10.1093/hr/uhac043)
Supplement: Web_Material_uhac043 [file web_material_uhac043.zip › Supplementary_figures/Supplementary_Figure2.pdf]

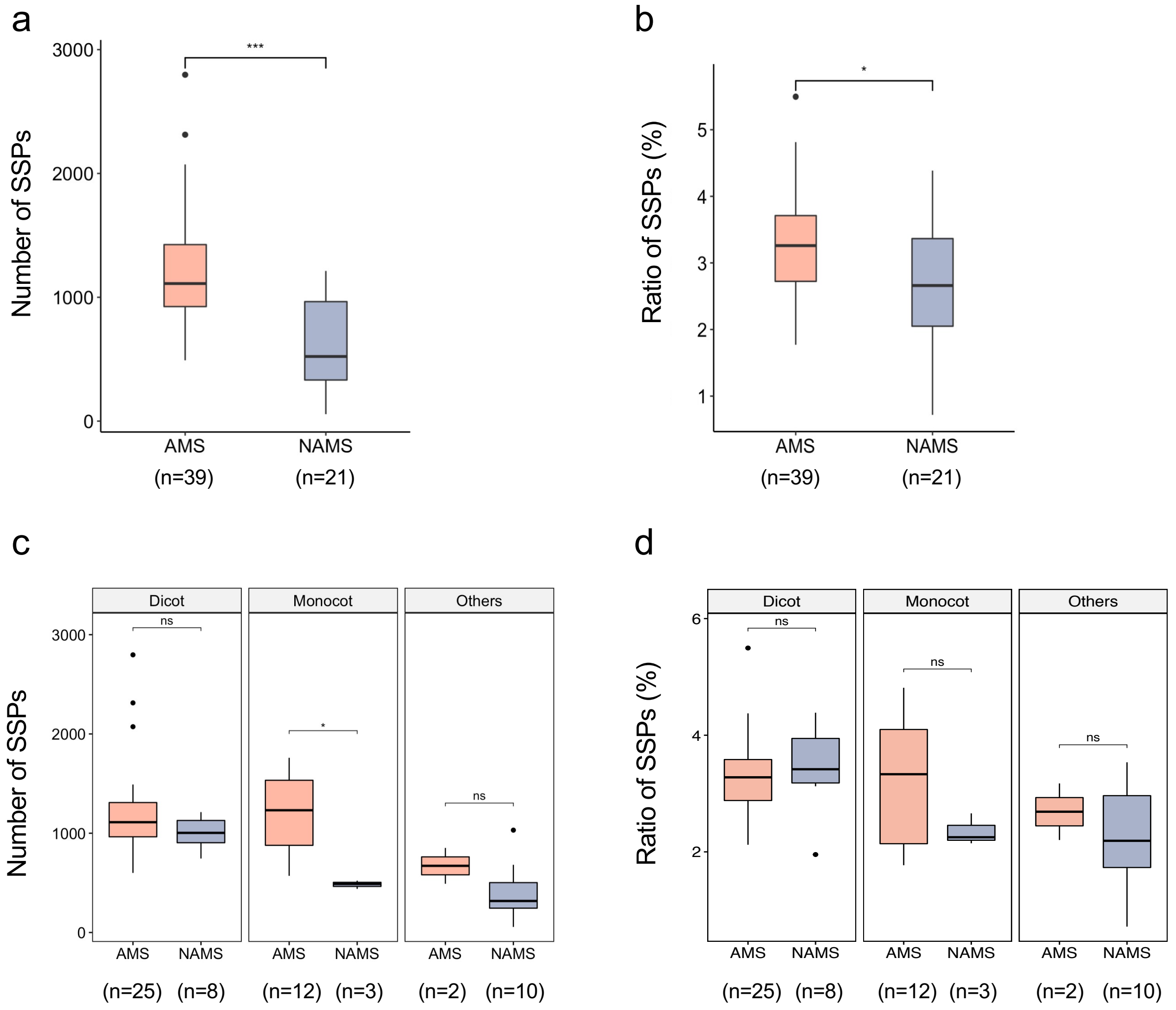

Supplementary Fig 2. Comparison of small secreted proteins (SSPs) in numbers and ratio between different plant species groups. The number of SSPs and the ratio they account in genomes were compared between AMS and non-AMS (NAMS) species using Wilcoxon-rank sum test with adjust $p < 0.05$ based on all plant species (a) and (b), both comparison show significant difference. Same strategy was also applied to plant groups containing dicots, monocots and other rest plant species, respectively (c) and (d). Only monocots showed significant difference in number of SSPs between AMS and NAMS species.
