# Supplementary material for: Diversity and conservation of plant small secreted proteins associated with arbuscular mycorrhizal symbiosis
Source: Hortic Res. 2022 Feb 19;9:uhac043. doi: 10.1093/hr/uhac043 (PMC8985099; doi:10.1093/hr/uhac043)
Supplement: Web_Material_uhac043 [file web_material_uhac043.zip › Supplementary_figures/Supplementary_Figure6.pdf]

(a)

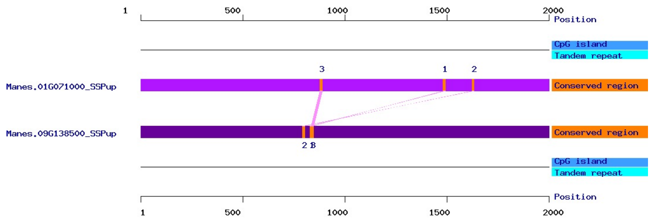

	TF ID (Gene ID)	Gene name (TF name)	TF Family	Species
1	AT1G09530	POC1; PIF3; PAP3	bHLH	Arabidopsis thaliana
2	AT1G29160		Dof	Arabidopsis thaliana
3	POPTR_0002s10560		AT-Hook	Populus trichocarpa

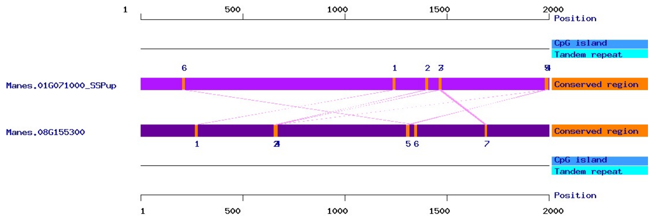

	TF ID (Gene ID)	Gene name (TF name)	TF Family	Species
1	AT4G14465	AHL20	AT-Hook	Arabidopsis thaliana
2	AT1G14900	HMGA	AT-Hook	Arabidopsis thaliana
3	BRADI5G22430	BRADI5G22430	AT-Hook	Brachypodium distachyon
4	AT1G14900	HMGA	AT-Hook	Arabidopsis thaliana
5	AT1G14900	HMGA	AT-Hook	Arabidopsis thaliana
6	AT5G16560	KAN; KAN1	Myb/SANT; MYB; G2-like	Arabidopsis thaliana
7	BRADI5G22430	BRADI5G22430	AT-Hook	Brachypodium distachyon

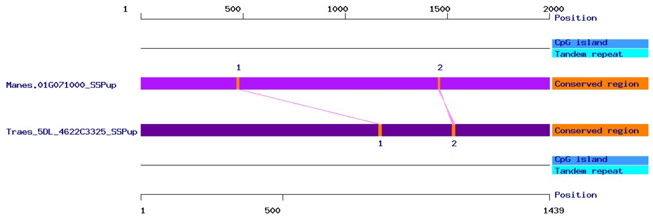

	TF ID (Gene ID)	Gene name (TF name)	TF Family	Species
1	AT5G06950	TGA2; AHBP-1B	bZIP	Arabidopsis thaliana
2	BRADI5G22430	BRADI5G22430	AT-Hook	Brachypodium distachyon

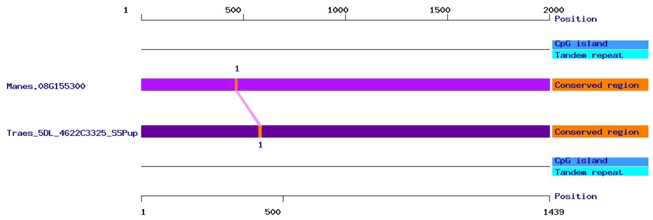

	TF ID (Gene ID)	Gene name (TF name)	TF Family	Species
1	AT3G61890	ATHB-12; ATHB12; HB-12	Homeodomain; bZIP; HD-ZIP	Arabidopsis thaliana

(b)

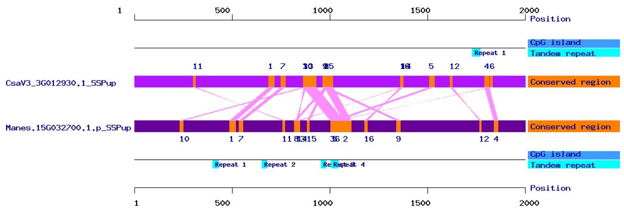

	TF ID (Gene ID)	Gene name (TF name)	TF Family	Species
1,2,3,4,5, 6			AT-Hook	
7	AT3G15500	NAC3; ATNAC3; ANAC055; NAC055	NAC; NAM	Arabidopsis thaliana
8	AT2G46830	CCA1	MYB-related	Arabidopsis thaliana
9	AT2G46830	CCA1	MYB-related	Arabidopsis thaliana
10,11			AT-Hook	
12	AT1G51600	TIFY2A; ZML2; GATA28	GATA	Arabidopsis thaliana
13			AT-Hook	
14	PK22848.1	PK22848.1	TCR	Cannabis sativa
15	AT2G46830	CCA1	MYB-related	Arabidopsis thaliana
16	PK22848.1	PK22848.1	TCR	Cannabis sativa

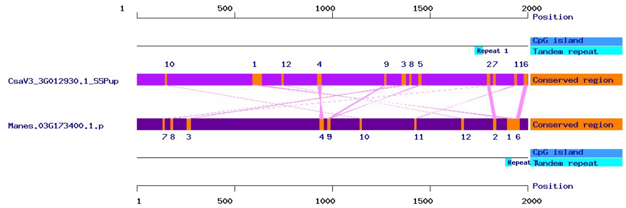

	TF ID (Gene ID)	Gene name (TF name)	TF Family	Species
1	BRADI5G22430	BRADI5G22430	AT-Hook	Brachypodium distachyon
2	AT1G69490	NAP; ANAC029; ATNAP	NAC; NAM	Arabidopsis thaliana
3	PK22848.1	PK22848.1	TCR	Cannabis sativa
4	POPTR_0002s10560	B9GN38_POPTR	AT-Hook	Populus trichocarpa
5	P38564	dof1	Dof	Zea mays
6	fgenesh2_kg.5__353__ AT3G25990.1	XM_002876896.1	MADF	Arabidopsis lyrata
7	AT4G35390	AHL25; AGF1	AT-Hook	Arabidopsis thaliana
8	AT4G35390	AHL25; AGF1	AT-Hook	Arabidopsis thaliana
9	AT1G63480	AHL12	AT-Hook	Arabidopsis thaliana
10	AT1G63480	AHL12	AT-Hook	Arabidopsis thaliana
11	e_gw1.01.00.699.1	e_gw1.01.00.699.1	MADS box	Ostreococcus tauri
12	Os01g0835600		AT-Hook	Oryza sativa

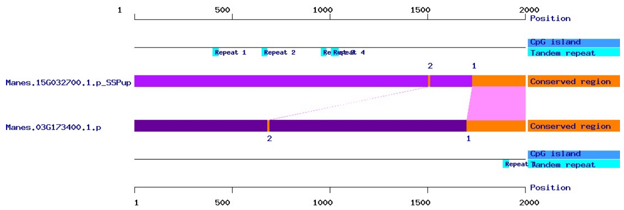

	TF ID (Gene ID)	Gene name (TF name)	TF Family	Species
1	AT3G04060	anac046	NAC; NAM	Arabidopsis thaliana
2	AT3G22760	SOL1	TCR; CPP	Arabidopsis thaliana

Supplementary Fig 6. Results of promoter alignment and *cis*-element identification. Representative genes in orthologue groups OG0000049(a) and OG0000364(b) were used to promoter alignment. *Cis*-elements were listed in the tables based on the number marked in the legends correspondingly.
