# Supplementary figures and images for: Diversity and conservation of plant small secreted proteins associated with arbuscular mycorrhizal symbiosis
Source: Hortic Res. 2022 Feb 19;9:uhac043. doi: 10.1093/hr/uhac043 (PMC8985099; doi:10.1093/hr/uhac043)

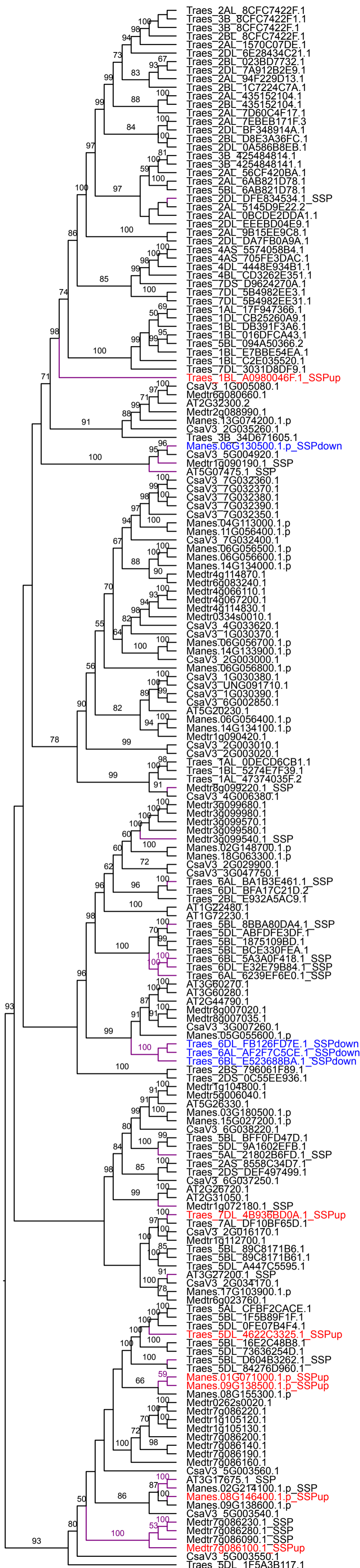

Supplement: Web_Material_uhac043 [file web_material_uhac043.zip › Supplementary_figures/Supplementary_Figure3.pdf]

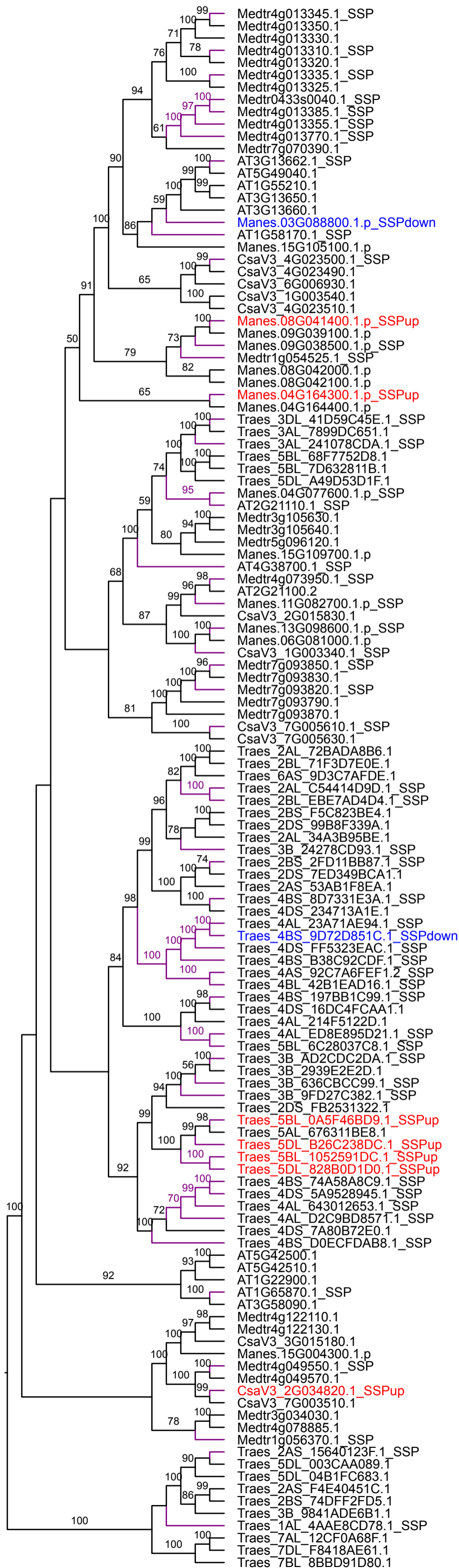

Supplement: Web_Material_uhac043 [file web_material_uhac043.zip › Supplementary_figures/Supplementary_Figure4.pdf]

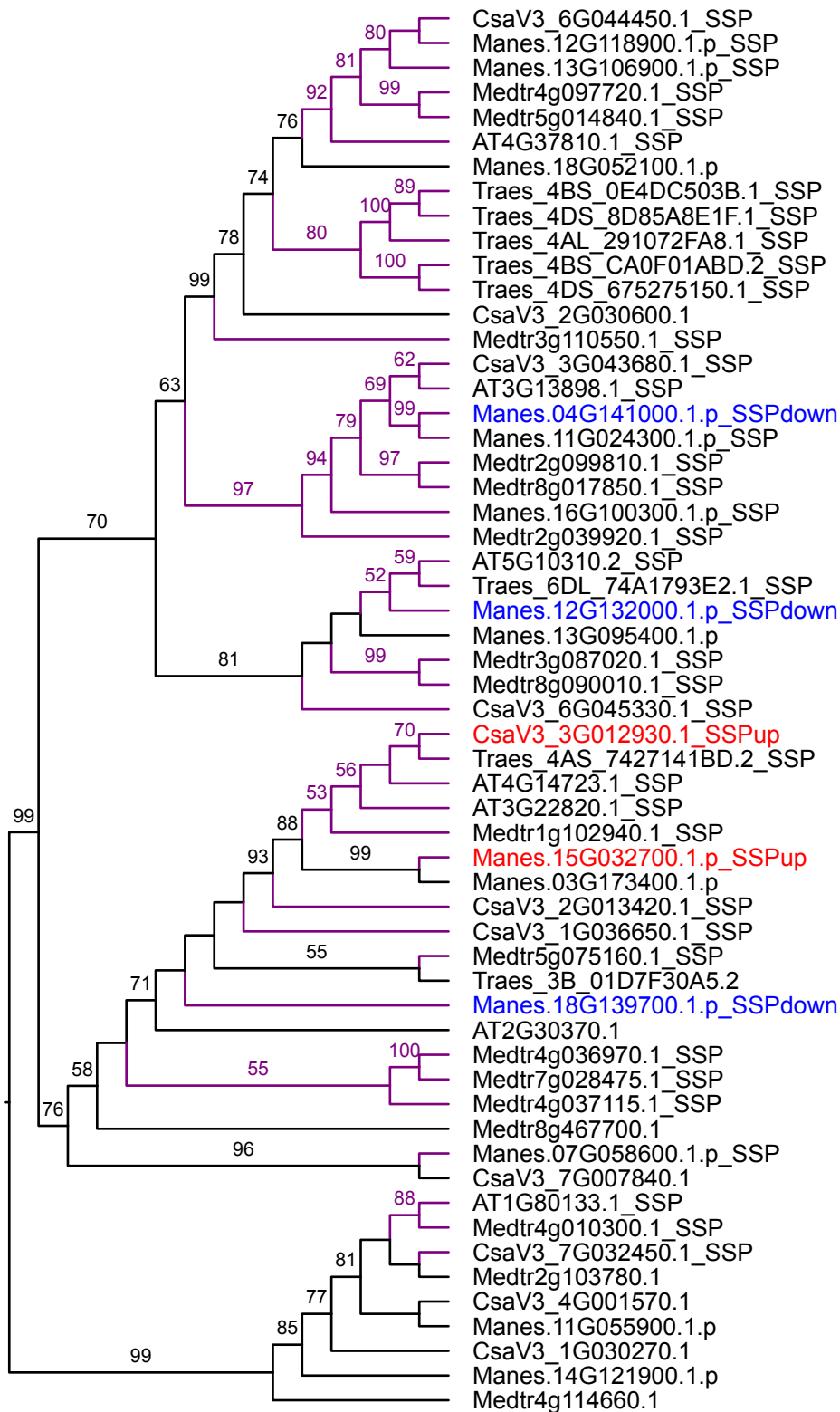

Supplement: Web_Material_uhac043 [file web_material_uhac043.zip › Supplementary_figures/Supplementary_Figure5.pdf]
